# Supplementary material for: Carbutamide, an Obsolete Anti-Diabetic Drug, Has Potential as a Potent Anticolitic Agent via Azo-Conjugation with Mesalazine
Source: Pharmaceutics. 2025 Nov 22;17(12):1509. doi: 10.3390/pharmaceutics17121509 (PMC12736148; doi:10.3390/pharmaceutics17121509)
Supplement: Supplementary file 1 [file pharmaceutics-17-01509-s001.zip › pharmaceutics-3912310-supplementary.pdf]

pharmaceutics

Supplementary Materials: Carbutamide, an obsolete anti-diabetic drug, has potential as a potent anticolitic agent via azo-conjugation with mesalazine

Sanghyun Ju, Suji Kim, Taeyoung Kim, Jin-Wook Yoo, In-Soo Yoon, Eunsoo Kim and Yunjin Jung

Supplementary Information S1. A preliminary experiment for UV absorption analysis for CBT and CAA Everted jejunal sacs were prepared as described in the main text and filled with pre-warmed Krebs buffer only (without drugs). The sacs were incubated at 37°C, and 50 μL samples were collected from the basolateral compartment at 10, 20, 30, 40, 60, and 90 min. The absorbance of each time-point sample was measured to confirm that background signals from tissue and buffer reached equilibrium. Collected samples were mixed with 575 μL methanol to precipitate proteins, followed by centrifugation at 10,000 × g for 10 min at 4°C. The resulting supernatants were used for UV measurement at λmax = 270 nm for CBT and λmax = 355 nm for CAA. The medium collected at 10 min was used as the blank for subsequent transport assays. During the main CBT and CAA transport experiments, sample absorbance values were corrected by subtracting this blank.

Supplementary Information S2. Assessment of intestinal barrier integrity in the Jejunum**.** To verify that the drug transport was not due to epithelial barrier disruption, intestinal barrier integrity was evaluated using FITC-dextran as a paracellular flux marker under the same experimental conditions as the transport study. Everted jejunal sacs were prepared as described in the main text. The basolateral side of each sac was filled with 600 μL of Krebs buffer, while the apical side contained 8 mL of the same buffer. FITC–dextran (100 μM) was added to the apical side together with either CAA (1 mM) or CBT (1 mM). The sacs were incubated at 37 °C. Samples (50 μL) were collected from the basolateral side at 10, 20, 30, 40, 60, and 90 min and immediately replaced with an equal volume of fresh Krebs buffer to maintain a constant volume. The collected samples were mixed with 575 μL of methanol to precipitate proteins, followed by centrifugation. The supernatant was used for fluorescence analysis. Fluorescence intensity was measured using a fluorescence spectrophotometer (excitation 495 nm, emission 520 nm). The apparent transport was expressed as the percentage of the FITC–dextran concentration on the basolateral side relative to the initial concentration on the apical side.

Supplementary Information S3. Assessment of intestinal barrier integrity in the Distal Colon.

To verify that the reduction in drug transport was not due to epithelial barrier disruption, intestinal barrier integrity was evaluated using FITC-dextran as a paracellular flux marker under the same experimental conditions as the transport study. Everted distal colonic sacs were prepared as described in the main text. The basolateral side of each sac was filled with 600 μL of Krebs buffer, while the apical side contained 8 mL of the same buffer. FITC–dextran (100 μM) was added to the apical side together with either CBT (5 mM), 5-ASA (5 mM), or a combination of CBT and 5-ASA (each at 5 mM). The sacs were incubated at 37 °C. Samples (50 μL) were collected from the basolateral side at 10, 20, 30, 40, 60, and 90 min and immediately replaced with an equal volume of fresh Krebs buffer to maintain a constant volume. The collected samples were mixed with 575 μL of methanol to precipitate proteins, followed by centrifugation. The supernatant was used

for fluorescence analysis. Fluorescence intensity was measured using a fluorescence spectrophotometer (excitation 495 nm, emission 520 nm). For the 5-ASA and 5-ASA + CBT groups, the samples were analyzed at 490 nm using a UV–Vis spectrophotometer due to interference with fluorescence measurement. The apparent transport was expressed as the percentage of the FITC–dextran concentration on the basolateral side relative to the initial concentration on the apical side.

Supplementary Information S4. Treatment groups for two independent animal experiments.

In the first experiment, rats were randomly assigned into six groups (n = 5 per group) and received the following oral treatments via gavage: group 1, normal control, administered 1.0 mL of PBS; group 2, colitis control, received 1.0 mL of PBS; group 3, SSZ-treated colitis group, administered SSZ (30.0 mg/kg) in 1.0 mL of PBS; group 4, mix-treated colitis group, administered a mixture of CBT (20.4 mg/kg) and 5-ASA (11.5 mg/kg) in 1.0 mL of PBS; group 5, low-dose CAA-treated colitis group, administered CAA (15.8 mg/kg, equivalent to 10.2 mg/kg of CBT) in 1.0 mL of PBS; and group 6, high-dose CAA-treated colitis group, received CAA (31.5 mg/kg, equivalent to 20.4 mg/kg of CBT, molar equivalent to 30.0 mg/kg of SSZ). For the second experiment, rats were randomly assigned into five groups (n = 5 per group) and received the following treatments via rectal route: group 1, normal control, administered 0.5 mL of PBS; group 2, colitis control, received 0.5 mL of PBS; group 3, 5-ASA-treated colitis group, administered 5-ASA (30.0 mM) in 0.5 mL of PBS; group 4, CBT-treated colitis group, administered CBT (30.0 mM) in 0.5 mL of PBS; group 5, Mix-treated colitis group, administered a mixture of CBT (30.0 mM) and 5-ASA (30.0 mM) in 0.5 mL of PBS. Three days after induction of colitis with DNBS, rats received once-daily oral or rectal administration of drugs for six consecutive days. The rats were euthanized 24 h after the final dose.

Table S1. Change in total amount of drugs during assays.

Treatment	Time (min)	% of initial amount
CBT	10	98.6
	40	98.5
	90	98.5
CAA	10	99.0
	40	98.9
	90	98.6
VCV	10	99.7
	40	99.3
	90	99.0

Amounts of drugs quantified in the apical and basolateral side (Figures 2A and 5) were combined to monitor change in the total amount of CBT, CAA, and VCV during assays.

Table S2. Exact *p*-values corresponding to the statistical analyses presented in Figure 3.

Figure	Comparison	*p*-value
Figure 3B	DNBS vs SSZ	$p = 0.048$
Figure 3B	DNBS vs CAA (L)	$p = 0.029$
Figure 3B	DNBS vs CAA (H)	$p = 0.029$
Figure 3B	SSZ vs CAA (H)	$p = 0.012$
Figure 3D	DNBS vs SSZ	$p = 0.003$
Figure 3D	DNBS vs CAA (L)	$p = 0.009$
Figure 3D	DNBS vs CAA (H)	$p < 0.001$
Figure 3D	SSZ vs CAA (H)	$p = 0.005$
Figure 3E	DNBS vs SSZ	$p = 0.038$
Figure 3E	DNBS vs CAA (L)	$p = 0.004$
Figure 3E	DNBS vs CAA (H)	$p = 0.001$
Figure 3E	SSZ vs CAA (H)	$p = 0.035$

Table S3. Exact *p*-values corresponding to the statistical analyses presented in Figure 4.

Figure	Comparison	*p*-value
Figure 4B	DNBS vs 5-ASA	$p = 0.029$
Figure 4B	DNBS vs Mixture	$p = 0.029$
Figure 4B	5-ASA vs Mixture	$p = 0.029$
Figure 4D	DNBS vs 5-ASA	$p = 0.003$
Figure 4D	DNBS vs CBT	$p < 0.001$
Figure 4D	DNBS vs Mixture	$p < 0.001$
Figure 4D	5-ASA vs Mixture	$p = 0.035$
Figure 4E	DNBS vs 5-ASA	$p = 0.009$
Figure 4E	DNBS vs CBT	$p = 0.027$
Figure 4E	DNBS vs Mixture	$p < 0.001$
Figure 4E	5-ASA vs Mixture	$p = 0.012$

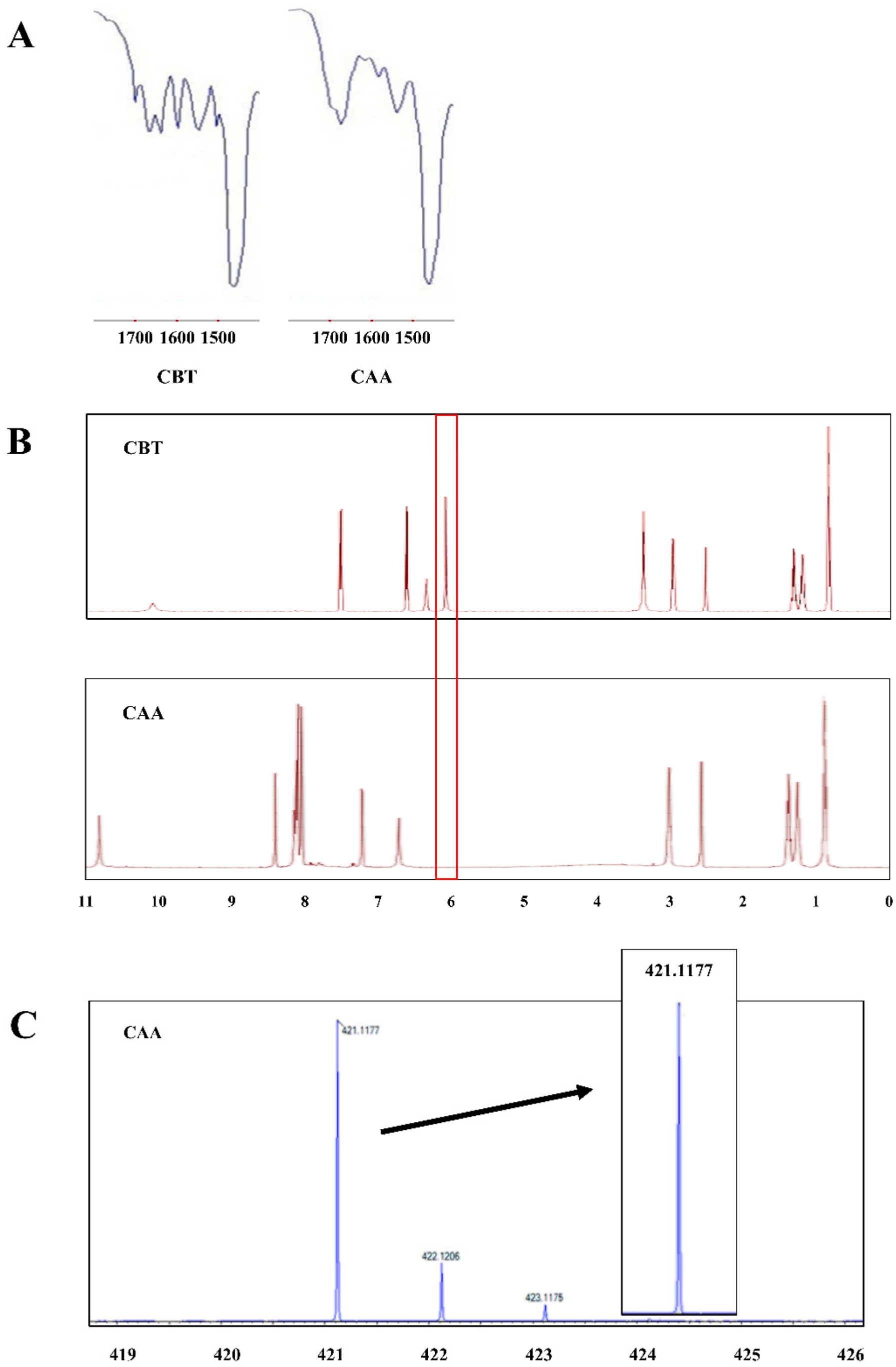

Figure S1. (A) FT-IR spectra of CBT and CAA. (B) ^{1}H-NMR spectra of CBT and CAA; the red box in the spectra indicates that the proton peak corresponding to the aromatic amine in CBT disappears in the spectrum of CAA. (C) Mass spectrum of CAA. CBT: carbutamide, CAA: CBT azo-linked with salicylic acid

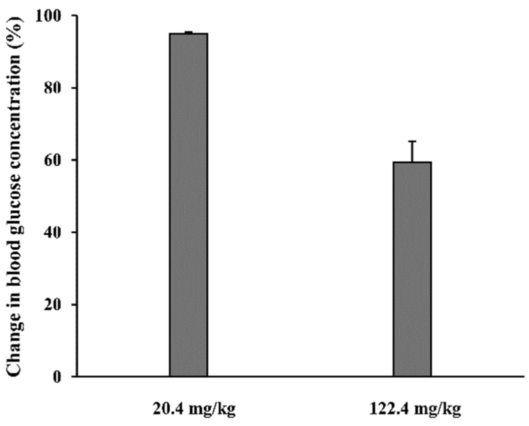

Figure S2. Rats were fasted for 18 h, followed by oral administration of CBT (20.4 mg/kg or 122.4 mg/kg), and blood glucose levels were measured at 5 h post-dose.
